# Coordinates of Human Visual and Inertial Heading Perception

**DOI:** 10.1371/journal.pone.0135539

**Published:** 2015-08-12

**Authors:** Benjamin Thomas Crane

**Affiliations:** 1 Department of Otolaryngology, University of Rochester, Rochester, NY, United States of America; 2 Department of Bioengineering, University of Rochester, Rochester, NY, United States of America; 3 Department of Neurobiology and Anatomy, University of Rochester, Rochester, NY, United States of America; University of Alberta, CANADA

## Abstract

Heading estimation involves both inertial and visual cues. Inertial motion is sensed by the labyrinth, somatic sensation by the body, and optic flow by the retina. Because the eye and head are mobile these stimuli are sensed relative to different reference frames and it remains unclear if a perception occurs in a common reference frame. Recent neurophysiologic evidence has suggested the reference frames remain separate even at higher levels of processing but has not addressed the resulting perception. Seven human subjects experienced a 2s, 16 cm/s translation and/or a visual stimulus corresponding with this translation. For each condition 72 stimuli (360° in 5° increments) were delivered in random order. After each stimulus the subject identified the perceived heading using a mechanical dial. Some trial blocks included interleaved conditions in which the influence of ±28° of gaze and/or head position were examined. The observations were fit using a two degree-of-freedom population vector decoder (PVD) model which considered the relative sensitivity to lateral motion and coordinate system offset. For visual stimuli gaze shifts caused shifts in perceived head estimates in the direction opposite the gaze shift in all subjects. These perceptual shifts averaged 13 ± 2° for eye only gaze shifts and 17 ± 2° for eye-head gaze shifts. This finding indicates visual headings are biased towards retina coordinates. Similar gaze and head direction shifts prior to inertial headings had no significant influence on heading direction. Thus inertial headings are perceived in body-centered coordinates. Combined visual and inertial stimuli yielded intermediate results.

## Introduction

Human heading estimation is multi-sensory involving visual and inertial cues[1–3]. However these sensory modalities use different reference frames with vision represented relative to the retina[4–6], vestibular relative to the head[6–9], and somatosensation relative to the body[10, 11]. It has been proposed that multisensory integration should occur in a common reference frame[12–15]. However, a single reference frame may be implausible based on recent findings for visual-vestibular integration[6, 16] as well as visual-proprioceptive[17] and auditory[18] integration which suggests multiple references frames.

Although the coordinates of perceived heading estimates have not previously been directly measured several studies have looked at the neurophysiology underlying this perception. The ventral intraparietal area (VIP) is a region that is likely to be importation for visual-vestibular integration[1, 19–22]. In VIP visual stimuli are represented in eye-centered coordinates[23, 24] while vestibular headings in VIP are in body coordinates that do not vary with changes in eye or head position[16]. In contrast, in both the dorsal medial superior temporal area (MSTd) and parietoinsular vestibular cortex vestibular headings are more head centered[16]. Although, the neurophysiology has not eliminated the possibility of a common coordinate system for perception of visual and vestibular headings, no such common coordinate system has been found as visual headings have only been found to be represented in retinal coordinates and vestibular headings have been found in only head and body coordinates.

It has recently been shown that human heading estimates are systematically biased so that lateral component is overestimated with both visual and vestibular stimuli[25, 26]. This behavior can be predicted by a population vector decoder (PVD) model based on a relatively larger number of units with sensitivity to lateral motion in MSTd[27]. However previously studies did not attempt to measure the effect of eye and head position on these biases.

In the current experiment, human visual and inertial head estimates were measured while systematically varying eye and head position. This experiment was designed to address two current controversies: First, determine the coordinate systems in which visual and vestibular stimuli are perceived. Second, determine if multisensory visual-vestibular integration occurs in a common coordinate system. Perception of visual headings shifted with gaze position demonstrating visual headings were perceived in retina-centered coordinates. Inertial heading estimates were not influenced by either head or eye position indicating a body-centered coordinates. When both visual and vestibular stimuli are present an intermediate coordinate system was used.

## Methods

### Ethics Statement

The research was conducted according to the principles expressed in the Declaration of Helsinki. Written informed consent was obtained from all participants. The protocol and written consent form were approved by the University of Rochester Research Science Review Board (RSRB).

### Equipment

Motion stimuli were delivered using a 6-degree-of-freedom motion platform (Moog, East Aurora, NY, model 6DOF2000E) similar to that used in other laboratories for human motion perception studies [26, 28–30] and previously described in the current laboratory for heading estimation studies[25, 31].

Head and platform movements were monitored in all six-degrees of freedom using a flux-gate magnetometer (trakSTAR, Ascension Technologies, Burlington, VT) using two model 800 position sensors, one on the subject’s head and other on the chair as previously described[32].

Monocular eye position was monitored and recorded at 60 Hz using an infrared video eye tracking system (LiveTrack, Cambridge Research Systems, Rochester England). Prior to each experiment the position was calibrated using a series of fixation points between ±30° in the horizontal plane and ±15° in the vertical plane. This system was used predominately as test of fixation, although failures of fixation were found to be extremely rare.

During both visual and vestibular stimuli, an audible white noise was reproduced from two platform-mounted speakers on either side of the subject as previously described [33]. The noise from the platform was similar regardless of motion direction. Tests in the current laboratory previously demonstrated that subjects could not predict the platform motion direction based on sound alone[33].

Sounds from the platform were further masked using a white noise stimulus reproduced from two platform-mounted speakers on either side of the subject. The intensity of the masking noise used in the current study varied with time as a half-sine wave so that the peak masking noise occurred at the same time the peak velocity was reached. This created a masking noise similar to the noise made by the platform. Sound levels at the location of the subject were measured using a Quest Technologies, model 1900 sound level meter (Quest Technologies, Oconomowoc, WI). Average sound pressure level (SPL) of the ambient sound was 58 dB, with a peak level of 68 dB when no motion was delivered. The masking noise had a peak of 92 dB. The motion platform had a peak noise level of 84 dB for velocities of 30 deg or cm/s for movements in the horizontal plane (yaw, surge, and sway) and 88 dB for heave. The peak noise of the platform was 74 dB. The masking sound intensity was the same for every stimulus independent of the stimulus direction and masking was also used for visual stimuli for consistency even though the visual stimulus made no sound. No masking noise was used between stimuli. We found this type of masking much more effective than a continuous masking noise of constant intensity.

Responses were collected using a two-button control box with a dial in the middle that could be freely rotated in the horizontal plane without any discontinuity points as previously described in the current laboratory[25].

### Stimulus

There were three types of stimuli: visual only, inertial only, and combined visual-inertial. During the combined stimulus condition the visual and inertial motion were synchronous and represented the same direction and magnitude of motion. The visual and inertial stimuli consisted of a single cycle 2s (0.5 Hz) sine wave in acceleration. This motion profile has previously been used for threshold determination[29, 33, 34] and for heading estimation[25, 31]. Directions tested included the 360° range of headings in 5° increments (72 total), delivered in random order. The displacement of the stimulus was 16 cm with a peak velocity of 16 cm/s and peak velocity of 25 cm/s/s.

Visual stimuli were presented on a color LCD screen (Samsung model LN52B75OU1FXZA) with a resolution of 1920 x 1080 pixels 50 cm from the subject filling 98° horizontal field of view. A fixation point consisted of a 2x2 cm cross at eye level could be presented centered or ±28° and was visible throughout every trial. The visual stimulus consisted of a star field which simulated movement of the observer through a random-dot cloud with binocular disparity as previously described[25]. Each star consisted of a triangle 0.5 cm in height and width at the place of the screen adjusted appropriately for distance. The star density was 0.01 per cubic cm. The depth of the field was 130 cm (the nearest stars were 20 cm and the furthest 150 cm). Visual coherence was fixed at 100%. Disparity was provided using red-green anaglyph glasses made with Kodak (Rochester, NY) Wratten filters #29 (dark red) and #61 (deep green). The colors were adjusted such that the intensities of the two were similar when viewed through the respective filters and rejection ratio was better than ten fold.

### Experimental Procedure

Three stimulus conditions were used: Inertial motion in which the platform moved, visual motion in which the platform remained stationary but star field motion was present, and combined visual and inertial motion.

Subjects were instructed that each stimulus would be a linear motion in the horizontal plan. Prior to testing subjects were show how to orient a mechanical dial as previously described[25]. A few practice trials were conducted in the light prior to doing the experiment. Although no feedback was given during these trials, if they were making systematic errors such as identifying the direction of the star field motion rather than their direction through the star field such errors were corrected.

Four types of head/gaze variations were used, each in a separate block of trials: Head centered gaze centered (HCGC) in which the head and gaze remained fixed at the midline. This condition was essentially the same as a previously published study[25], but was repeated using the current subjects. In the remaining 3 types of trial blocks there were interleaved conditions in which head or gaze position was randomly varied between trials. The head remained fixed in the head centered gaze varied (HCGV) condition while gaze was varied between fixation points 28° to the right or left prior to each stimulus presentation. In the head varied, gaze centered (HVGC) condition the visual fixation point remained centered and the head was rotated to 28° to the right or left prior to each stimulus presentation. In the head varied, gaze varied condition (HVGV) both the head and gaze were rotated 28° to the right or left prior to the stimulus presentation such that the eye remained near straight ahead relative to the head.

In the HVGC and HVGV conditions prior to each trial (i.e. each stimulus presentation) a location representing the ideal head position (28° right or left) was shown on the video display. A box ±1° on a side represented the current head position in real time and the subject was instructed to move their head so that the cross was in the center of the box. Afterwards the head was stabilized against a rubber headrest. A fixation point was displayed to indicate gaze position which in the case of HVGV was the center of the box and in HVGC was in the center of the screen. Once the head and gaze were stabilized at the desired position the subject pressed the start button. After pressing the start button the box and current head location were no longer displayed but were recorded through out the trial. Immediately after the stimulus was delivered subjects heard two 0.125s tones in rapid succession to indicate their response could be reported using the dial. After they were finished they pushed a button to indicate they were done.

### Subjects

A total of 7 subjects (5 female) completed the 11 trial block protocol. Ages ranged from 20 to 67 (39 ± 22, mean ± SD). None of the subjects was familiar with the design of the experiment. The order of trial blocks was randomized between subjects and no feedback was given. To minimize fatigue the trials blocks were completed in multiple sessions on different days with 2–3 blocks completed in each session. All subjects were screened prior to participation and had normal or corrected vision, normal hearing, normal vestibular function on caloric testing, and no dizziness or balance symptoms.

### Analysis

Each dial setting was compared with the actual heading for each trial to calculate a response error. A simple population vector decoder (PVD) model was fit to each participants responses for each test condition. The general form of a PVD model is given in Eq (1).

$$\vec{P}=\sum_{i=1}^{n}w_{i}\vec{C_{i}}$$ (1)

Because there is little known about the actual distribution of human sensitivities, the model was simplified to include only two orthogonal vectors representing surge and sway with independent weights. Although including additional vectors in the population would allow more degrees of freedom and a better fit to the data, this was not done due to the risk of over fitting:

$$\vec{P}=w_{sway}{\vec{C}}_{sway}+w_{surge}{\vec{C}}_{surge}$$ (2)

The absolute weights are not important but only their relative sizes, so they can be replaced with a ratio:

$$r=\frac{w_{sway}}{w_{surge}}$$ (3)

This gives us:

$$\vec{P}=w_{surge}\left(r{\vec{C}}_{sway}+{\vec{C}}_{surge}\right)$$ (4)

**Fig 1:**
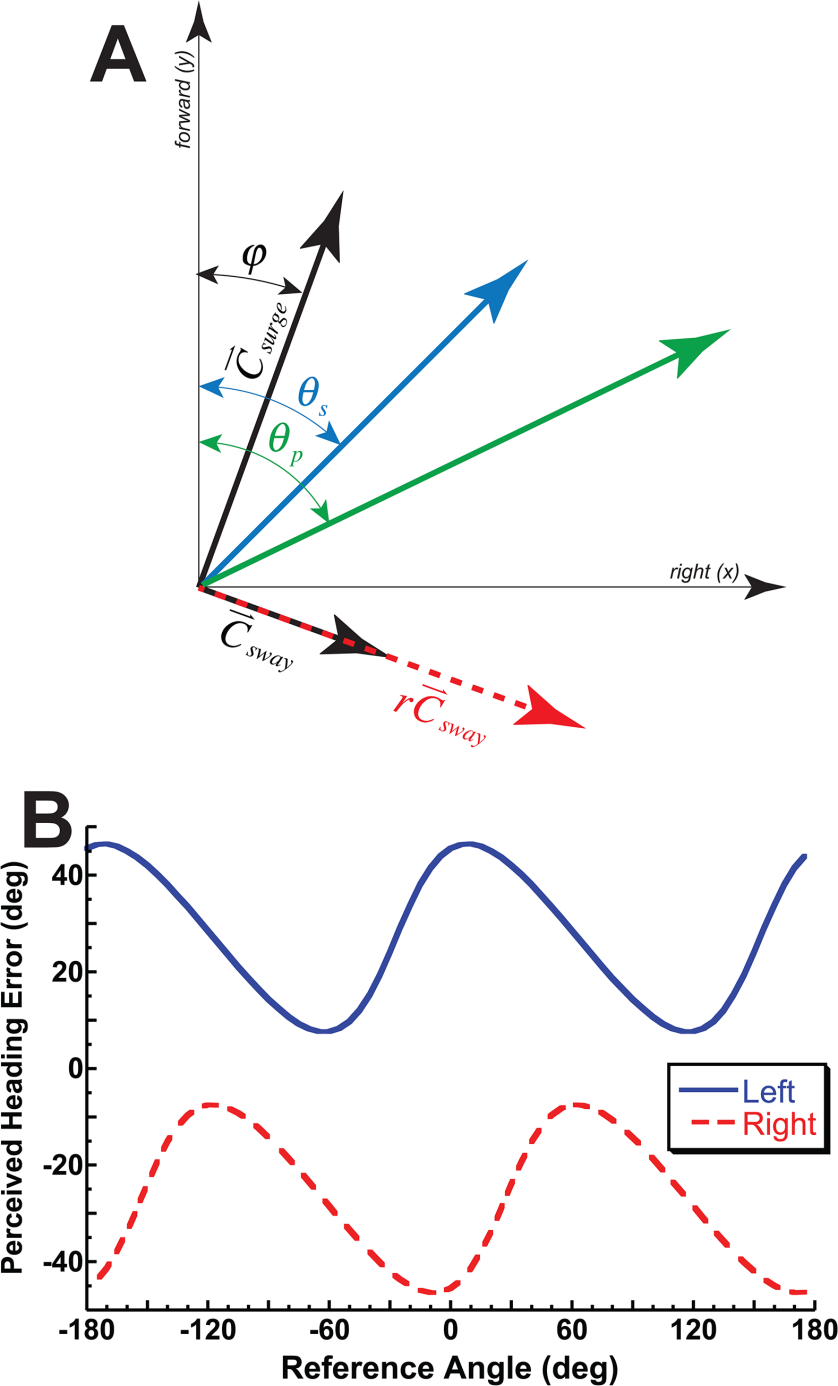
The population vector decoder (PVD) model used and its predictions. (A) The model in graphical form for a stimulus heading, *θ*_*s*_, of 45° to the right. This 45° vector can be represented by orthogonal surge, ${\vec{C}}_{surge}$, and sway, ${\vec{C}}_{sway}$, vectors offset by *φ*. The perceived heading, *θ*_*p*_, is the sum of the surge and sway vectors after the sway component is multiplied by *r*. (B) The quantitative predictions of the model when r = 2 (sway component weighted twice that of surge). The solid blue tracing represents a 27° leftward gaze and the dashed red tracing represents a 27° rightward gaze. Ideal performance would be a perceived heading error of 0° for all reference headings. The model predicts a gaze shift changes the coordinate system and influences both the phase and offset of the perceived heading error.

Because we are only interested in the direction of the vector and the length is not important the absolute weight of the surge component is not needed. This simplifies the PVD to (Fig 1A):

$$\vec{P}=r{\vec{C}}_{sway}+{\vec{C}}_{surge}$$ (5)

The orientation of the stimulus in space is given by *θ*_*s*_. The surge and sway vectors relative to space were allowed to vary by an offset angle or phase (*φ*). Here the perceived direction, $\vec{P}$, is related to the unit forward vection, $\hat{y}$, and unit sway vector, $\hat{x}$:

$$\vec{P}=r\sin\left(\theta_{s}+\varphi \right)\hat{x}+\cos\left(\theta_{s}+\varphi \right)\hat{y}$$ (6)

Thus when ${\vec{C}}_{surge}$ is forward (i.e. cos(*θ*_*s*_ + *φ*) > 0) the perceived heading angle, is given by:

$$\theta_{p}=\tan^{-1}\left(r\tan\left(\theta_{s}+\varphi \right)\right)$$ (7)

When ${\vec{C}}_{surge}$ is backwards and ${\vec{C}}_{sway}$ is to the right (i.e. sin(*θ*_*s*_ + *φ*) > 0) then a correction factor of +180° is applied, otherwise (i.e. sin(*θ*_*s*_ + *φ*) < 0) a correction of -180° is needed.

It is hypothesized that this PVD model can explain the perceived heading bias. This hypothesis is based on prior human behavior experiments[25, 26] as well as primate neurophysiology[27] which suggest greater numbers of units sensitive to changes in lateral or sway heading changes for both visual and inertial headings or in terms of the current model r > 1. If visual headings are perceived in retina coordinates as suggested by recordings from VIP[23, 24], then the offset angle (*φ*) will be similar to the eye position (Fig 1B). This model was fit to the responses for each condition. This was done using the fminsearch function in Matlab (Mathworks, Natick, Massachusetts) to minimize the mean squared error (MSE) of the model predictions relative to the observed responses.

Statistics was performed using JMP Pro version 11 for the Macintosh (SAS, Cary, North Carolina). A paired student’s t-test was used to determine significance between model fit parameters across the population tested. One way analysis of variance (ANOVA) was used to determine if there were significant effects of gaze/head position, stimulus types, and between subjects.

## Results

Eye position at the start of the trial was within 1° of the intended target. Eye fixation remained at the intended point during motion. In conditions in which the head position was varied over ±28°, the subjects were able to do this accurately. The average error was 0.2° at the start of the trial with the maximum <1°. The head also remained in position during the inertial movement. The average peak-to-peak variation in yaw head position during the inertial movement in head varied conditions was 1.9° with the standard deviation of head position averaging 0.6°.

**Fig 2:**
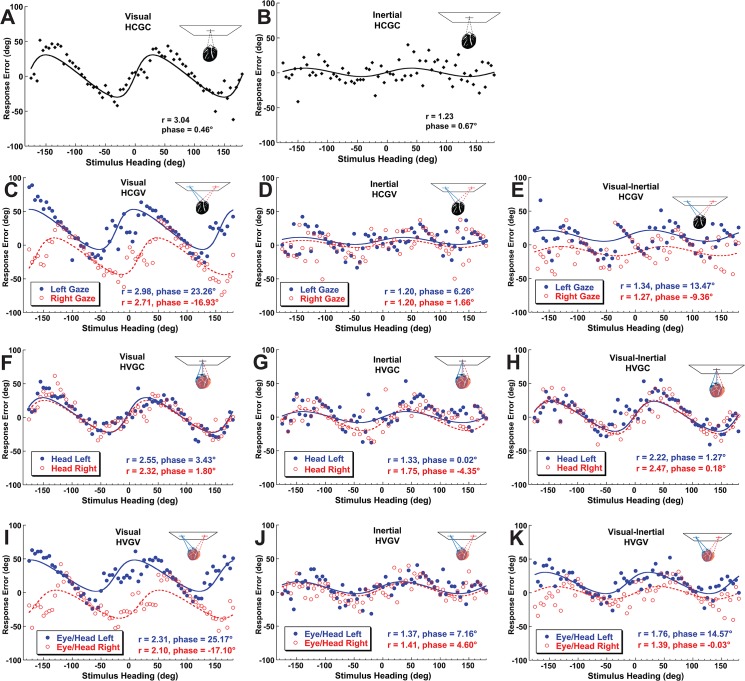
Sample data and model fits for a typical individual subject (#4). The abscissa represents the stimulus heading. The ordinate represents the response error (perceived minus stimulus heading). Curves represent the best fit of the PVD model. Top row (A&B): The head centered gaze centered (HCGC) condition. Second row (panels C-E): The head centered gaze varied (HCGV) condition with gaze varied by ±28°. The gaze direction strongly influenced visual heading perception as indicated by the PVD model phase/offset parameter (*φ*). Third row (F-H): Head position varied over ±28° with gaze centered (HVGC). Inertial headings were independent of head position. Bottom row (I-K): Varying gaze and head yielded results similar to varying gaze alone (C-E).

Performance of a typical subject (#4) is shown in Fig 2 with the results of model fit to the data. The visual headings (Fig 2A, 2C, 2F, & 2I) had heading specific biases that could be predicted by relative greater sensitivity to sway motion as the best fit ratio (r) ranged from 2.1 to 3. The heading offsets were a function of gaze position (Fig 2C & 2I). Inertial headings were predicted using a sensitivity to sway closer to that of surge with the best r fit ranging from 1.2 to 1.8 (Fig 2B, 2D, 2G, & 2J). Inertial heading perception was independent of gaze or head position. When visual and inertial headings were combined (Fig 2E, 2H, & 2K) the perceived headings and model fits represented a response that was intermediate between the visual and inertial only conditions.

**Fig 3:**
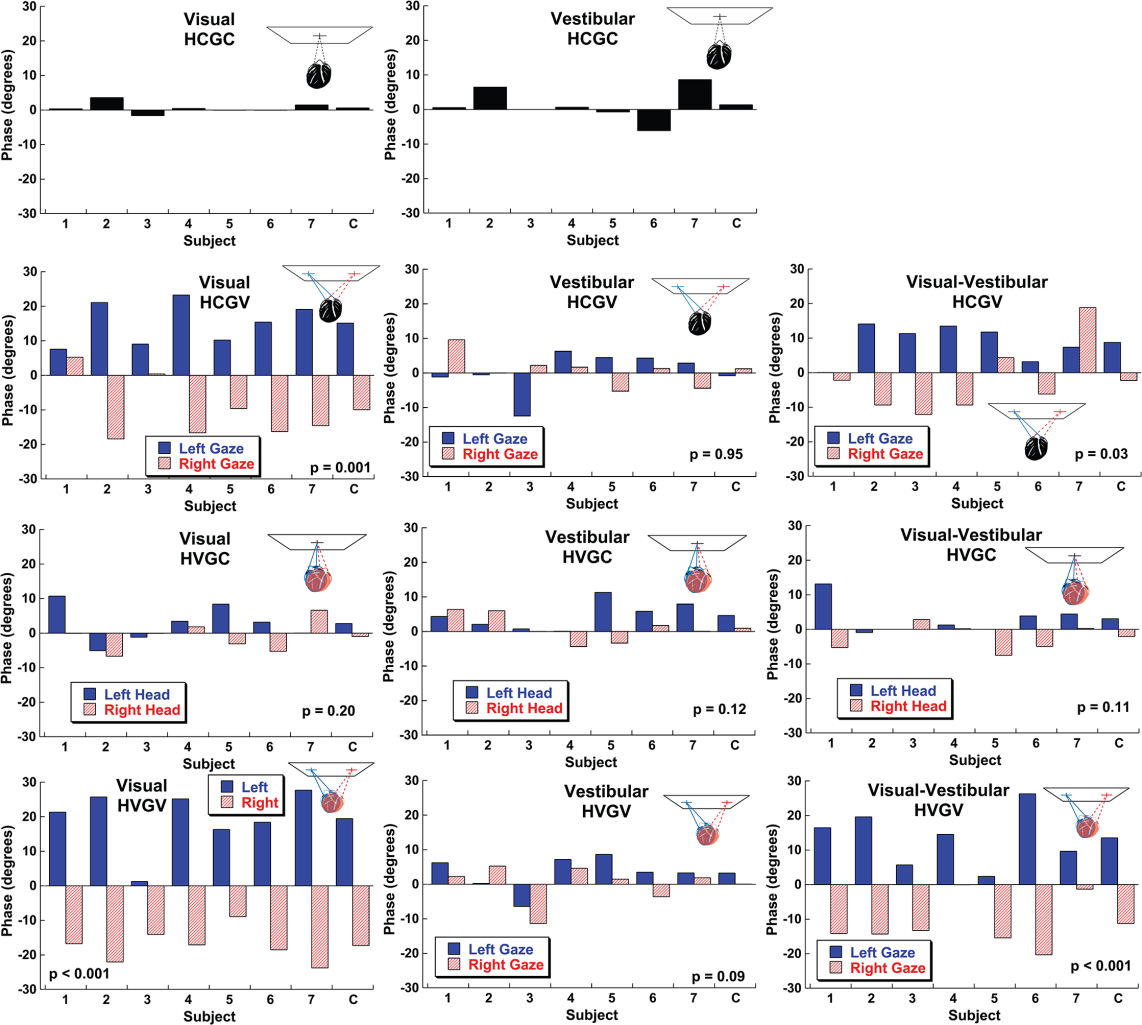
The phase/offset parameter (*φ*) of the PVD model for each of condition across subjects. The average (combined) value is shown in the furthest right column marked with a C. For the HCGC conditions the average phase offset was near zero. In conditions where the trial blocks included multiple head and/or gaze positions (panels C-K) a T-test was used to determine if the values were significantly different across subjects and p-values are printed for each condition.

The data were summarized using the ratio (*r*) and phase or offset (*φ*) parameters of the PVD model fit. The offset parameters that best fit each subject’s responses are shown for each subject and trial block type (Fig 3). For the HCGC conditions the offset averaged near zero (Fig 3A & 3B). In visual heading conditions where gaze was varied there was a large and significant effect of gaze direction (Fig 3C & 3I). The average offset was 13 ± 2° (mean ± SEM) in the direction of gaze or 46% of the gaze shift with the head centered and 17 ± 2° or 61% of the gaze shift in the HVGV condition. The mean effect was about half the size at 7.2 ± 1.4° (HCGV) and 12.8 ± 1.8° (HVGV) but remained highly significant with combined visual and inertial headings (Fig 3E & 3K). In every subject, when a visual stimulus was present, gaze shifted the perceived heading estimate so that left gaze produced a positive (rightward) offset and right gaze produced a relatively negative (leftward) offset. This was consistent with retina based coordinates since a leftward gaze would cause stationary objects to appear shifted to the right and vice-versa. Shifts in gaze did not cause a significant shift in inertial heading perception with the average shift being -0.3±1.4° (HCGV). Similarly head shifts (HVGC) produced a non-significant offset of 2.0 ± 1.2°.

**Fig 4:**
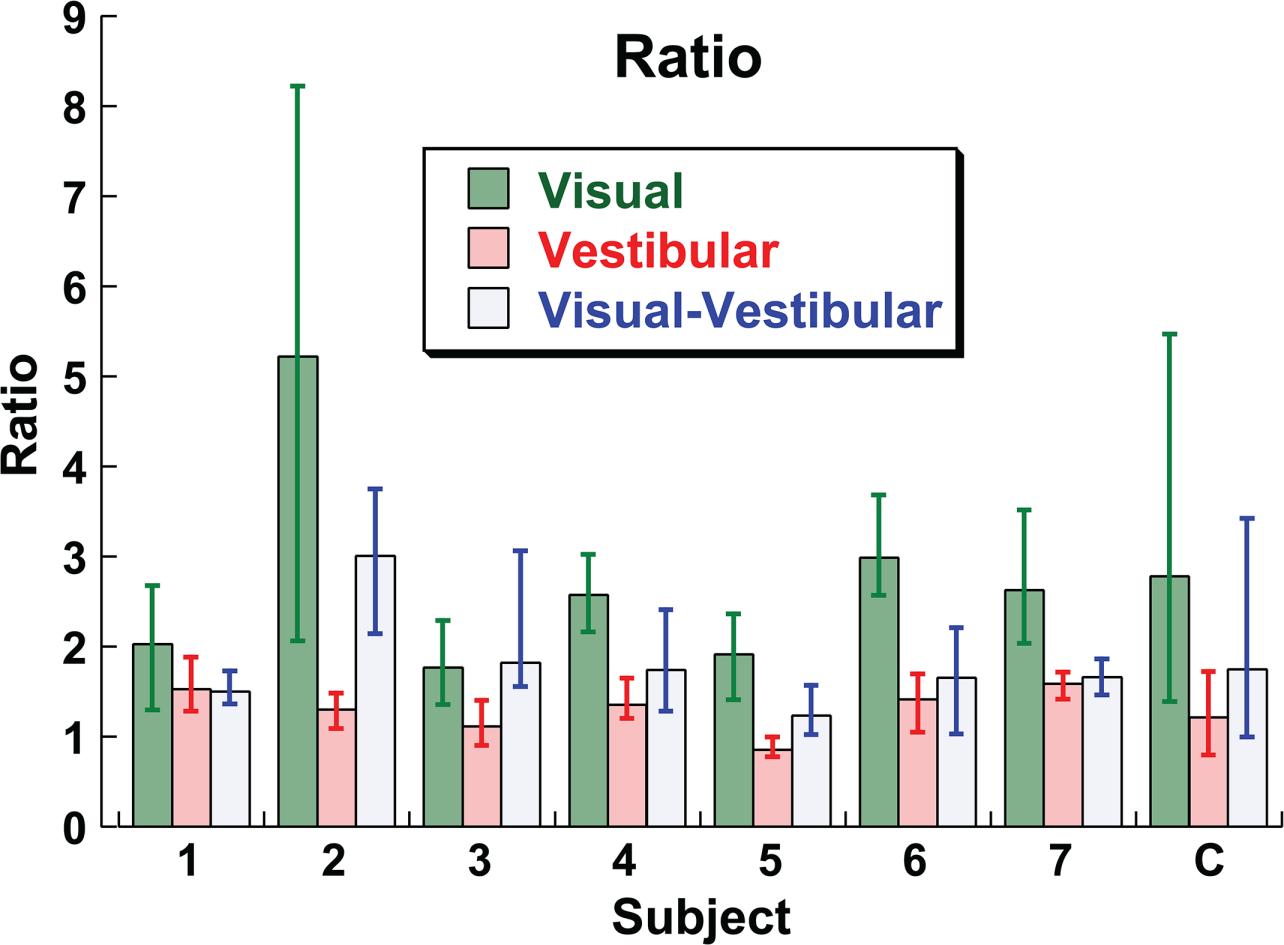
The ratio parameter (*r*) of the PVD model. Unlike the offset parameter, ratio was independent of gaze/head condition which where combined. The values combined across subjects are shown in the furthest right column marked C. Error bars represent the 95% CI.

Unlike the offset, the ratio (r) parameter of the model that best fit the data did not depend on the gaze or head position (e.g. HVGC, HCGV, etc.; ANOVA, p = 0.98, F = 0.06). The ratio depended on the stimulus type (e.g. visual, inertial, or combined; ANOVA p < 0.0001, F = 32.37). The ratio did not depend on the direction of gaze or head position (ANOVA p = 0.63, F = 0.23). The mean r for a visual stimulus was 2.78 (95% CI: 1.39–5.47), for an inertial stimulus it was 1.21 (95% CI: 0.79–1.72), for the combined visual-inertial stimulus it was 1.75 (95% CI: 0.99–3.42). There was significant variation in r across subjects (ANOVA p < 0.0001, F = 6.45). The ratio is shown by subject and stimulus type in Fig 4. In every subject the ratio was higher for the visual condition relative to the inertial condition. The combined stimulus yielded a ratio that had a variable relationship to the visual and inertial ratio depending on subject and could be closer to inertial (subjects 1, 6, 7), similar to visual (subject 3), or intermediate between the two (subjects 2, 4, 5).

Once fit with the ideal ratio and offset parameters the MSE of the fit of the model to the data did not depend on gaze/head condition (ANOVA, p = 0.72) or stimulus type (ANOVA, p = 0.52).

## Discussion

The method used here to measure heading estimates is a relatively simple task which has the potential to be influenced by confounding factors including cognitive influences. Yet, the large offsets in visual heading estimates with changes in gaze position were consistently observed across subjects and predicted from the known neurophysiology. In VIP, visual motion stimuli have been shown to be represented in eye-centered coordinates[23, 24]. The current data demonstrate that although visual headings are biased towards eye position, the bias in the heading was only about half of the eye position offset. This is likely because eye position is also considered in heading estimation[35–38], although the current data demonstrate that the effect of eye position is not completely corrected. Although it is possible that visual headings could be represented in body or world coordinates elsewhere in the central nervous system no such area has yet been identified. The current data suggest that no such representations of visual headings exist as subject’s reported perception of visual headings was strongly biased towards retina coordinates.

The other major finding was that inertial heading was represented in a body fixed coordinate system. Even large variations in head and gaze position had no influence on the heading estimates. The current experiments did not vary the body orientation relative to the earth so an earth fixed coordinate system is also possible. However, it is clear that the inertial headings and visual headings are perceived relative to different coordinate systems. This is consistent with the neurophysiology which demonstrates that in VIP, vestibular heading representation does not change with changes in either eye or head position[16]. Although it has been shown in MSTd and the parientoinsular vestibular cortex vestibular headings appear to be in head centered coordinates [6, 16]. It is not surprising that there are areas of the brain where inertial signals are represented in head coordinates as the vestibular organs are fixed in the head, but the current data demonstrates that inertial heading perception follows the neurophysiology of VIP most closely.

Previous studies have looked at multisensory integration using visual and inertial cues[3, 39–41]. A common model of multisensory integration is that cues are integrated in a statistically optimal way also known as an ideal observer model. This model predicts that each sensory cue will be weighted according to its relative reliability. This weighting strategy is what is predicted by Bayesian probability theory[42]. For visual-vestibular heading integration, the prior work in this area has focused on multisensory integration using a discrimination task (e.g. subjects report if a test stimulus is to the right or left of a reference heading). The current experiments involve a estimation task which allows the bias and reference frame to also be determined. The relative reliability of visual and inertial cues is more complex for heading estimation because the relative reliability varies not only on the stimulus but also with heading direction. The current experiments did not vary the relative reliabilities of the stimuli or offset the visual and inertial stimuli relative to each other which limits the scope of conclusions that can be made with regard to multi-sensory integration. However, the phase offsets (Fig 3) and ratios (Fig 4) calculated demonstrated that the combined condition was usually intermediate between the visual and inertial condition. In some subjects the combined stimulus was closer to inertial (e.g. subject 1) while in others it was similar to visual (e.g. subject 3). It seems clear that there is no uniform common reference frame for heading estimation, but how the intermediate reference frame is developed remains unclear.

An obvious issue raised by these results is that if eye position causes large biases in heading perception, what are the implications for day-to-day activities such as ambulation and driving? It is possible that feedback could minimize the biases observed here. The effect of feedback was not studied in the current experiments and it is likely that subjects were not aware that their responses were biased. However, these subjects also had feedback during their daily activities such as driving and ambulation that did not eliminate these biases. During ambulation people tend to direct gaze in the direction of intended motion[43, 44] which makes it easier to maintain an accurate heading[36] and this also occurs with driving[45–47]. Thus, under natural conditions, control of gaze direction may be the mechanism by which heading errors that could arise with eccentric gaze positions are minimized. When gaze is eccentric from the intended course by as little as 5° while driving, subjects shifted their position on road significantly toward the direction of gaze[47]. With vestibular headings in body coordinates, it is not surprising that head orientation changes do not influence ambulation direction [48]. The current data suggest fixed eccentric gaze while driving could lead to deviation towards the direction of gaze. Such an experiment has not to the author’s knowledge been done but, fixing gaze at a central position causes steering errors and decreased performance in driving simulation[49, 50]. Thus prior behavioral data is consistent with the current findings.

The current data strongly suggest that visual-vestibular heading estimation occurs in different reference frames.
